# 2-(4-Methoxy­phen­yl)-1*H*-indene

**DOI:** 10.1107/S1600536808019776

**Published:** 2008-07-05

**Authors:** Pu Liu, Zhen Liu, Xiao-Wei Wang, Wei Wang

**Affiliations:** a Chemical Engineering and Pharmaceutical College, Henan University of Science and Technology, Luoyang 471003, People’s Republic of China

## Abstract

Excluding four H atoms, the molecule of the title compound, C_16_H_14_O, is almost planar, with an r.m.s. deviation of 0.0801 (2) Å. Due to *p*–π conjugation, the lengths of the two single bonds attached to the O atom are significantly different.

## Related literature

For related literature, see: Rayabarapu *et al.* (2003 ▶); Senanayake *et al.* (1995 ▶).
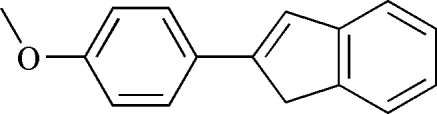


## Experimental

### 

#### Crystal data

C_16_H_14_O
*M* *_r_* = 222.27
Monoclinic, 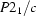

*a* = 5.8347 (8) Å
*b* = 7.5584 (10) Å
*c* = 26.135 (4) Å
β = 92.772 (11)°
*V* = 1151.3 (3) Å^3^
*Z* = 4
Mo *K*α radiation
μ = 0.08 mm^−1^
*T* = 113 (2) K
0.34 × 0.32 × 0.12 mm

#### Data collection

Rigaku Saturn diffractometer
Absorption correction: multi-scan (*CrystalClear*; Molecular Structure Corporation & Rigaku, 1999 ▶) *T*_min_ = 0.974, *T*_max_ = 0.991
10940 measured reflections
2724 independent reflections
2360 reflections with *I* > 2σ(*I*)
*R*_int_ = 0.038

#### Refinement

*R*[*F* ^2^ > 2σ(*F* ^2^)] = 0.051
*wR*(*F* ^2^) = 0.135
*S* = 1.10
2724 reflections
155 parameters
H-atom parameters constrained
Δρ_max_ = 0.32 e Å^−3^
Δρ_min_ = −0.39 e Å^−3^

### 

Data collection: *CrystalClear* (Molecular Structure Corporation & Rigaku, 1999 ▶); cell refinement: *CrystalClear*; data reduction: *CrystalClear*; program(s) used to solve structure: *SHELXS97* (Sheldrick, 2008 ▶); program(s) used to refine structure: *SHELXL97* (Sheldrick, 2008 ▶); molecular graphics: *SHELXTL* (Sheldrick, 2008 ▶); software used to prepare material for publication: *SHELXTL*.

## Supplementary Material

Crystal structure: contains datablocks global, I. DOI: 10.1107/S1600536808019776/wk2085sup1.cif

Structure factors: contains datablocks I. DOI: 10.1107/S1600536808019776/wk2085Isup2.hkl

Additional supplementary materials:  crystallographic information; 3D view; checkCIF report

## Figures and Tables

**Table 1:** Selected geometric parameters (Å, °)

O1—C1	1.3724 (16)
O1—C16	1.4301 (19)

**Fig. 1.**
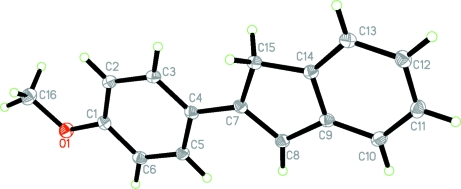
View of the molecule of (I) showing the atom-labelling scheme. Displacement ellipsoids are drawn at the 35% probability level.

## supplementary crystallographic information

### Comment

Indene ring frameworks are present in a large number of biologically active compounds, and their metallocene complexes are able to catalyze olefin polymerization (Senanayake *et al.*, 1995; Rayabarapu *et al.*, 2003). Some derivatives have shown analgesic and myorelaxation activity, and others are used as valuable intermediates for the synthesis of indenyl chrysanthemates that possess insecticidal properties. So in the recent three decades, many chemists have been attracted by the synthesis of indenes. In this context, we report the synthesis and crystal structure of the title compound, (I), namely 2-(4-methoxyphenyl)-1*H*-indene.

The title compound was obtained as colourless plate-like crystals in the monoclinic space group P 1 21/c 1. A view of the molecular structure of (I) with the numbering scheme is shown in Fig. 1. The whole molecular structure is almost planar with an r.m.s. deviation of 0.0801 (2) Å. Due to the *p*–π conjugation of atom O1 and benzene ring, the single-bond distance of the O1—C1 [1.3724 (16) Å] is significantly shorter than that of O1—C16 [1.4301 (19) Å].

### Experimental

*o*-Bromobenzyl zinc bromide (3.5 mmol, 3.5 equiv) in 3.5 ml CH_2_Cl_2_ was added to a degassed refluxing CH_2_Cl_2_ solution (8 ml) of 1-ethynyl-4-methoxybenzene (1.0 mmol, 1.0 equiv) and Ni(PPh_3_)_2_I_2_ (0.1 mmol, 0.1 equiv). After being stirred at 313 K for 6 h, the solution was cooled to room temperature. The resultant solution was diluted with 50 ml ethyl acetate. The organic layer was washed with 10 ml aqueous HCl solution, saturated NaCl. The aqueous layer was back-extracted with Ethyl acetate. The combined organic layer was dried over anhydrous Na_2_SO_4_. After filtration, the solvent was removed under reduced pressure and the residue was purified *via* flash chromatography (SiO_2_) to afford the compound. Single crystal suitale for X-ray analysis were obtained by slow evaporation at 298 K of a CH_2_Cl_2_ solution.

### Refinement

H atoms were positioned geometrically and refined as riding with C—H = 0.95–0.99 Å. For the CH and CH_2_ groups, *U*_iso_(H) values are set equal to 1.2*U*_eq_ (carrier atom) and for the methyl groups they are set equal to 1.5*U*_eq_ (carrier atom).

### Crystal data

**Table:**

C_16_H_14_O	*F*_000_ = 472
*M**_r_* = 222.27	*D*_x_ = 1.282 Mg m^−^^3^
Monoclinic, *P*2_1_/*c*	Mo *K*α radiation λ = 0.71070 Å
Hall symbol: -P 2ybc	Cell parameters from 2782 reflections
*a* = 5.8347 (8) Å	θ = 2.8–27.9º
*b* = 7.5584 (10) Å	µ = 0.08 mm^−^^1^
*c* = 26.135 (4) Å	*T* = 113 (2) K
β = 92.772 (11)º	Plate, colourless
*V* = 1151.3 (3) Å^3^	0.34 × 0.32 × 0.12 mm
*Z* = 4	

### Data collection

**Table:**

Rigaku Saturn diffractometer	2724 independent reflections
Radiation source: rotating anode	2360 reflections with *I* > 2σ(*I*)
Monochromator: confocal	*R*_int_ = 0.038
Detector resolution: 7.31 pixels mm^-1^	θ_max_ = 27.9º
*T* = 113(2) K	θ_min_ = 2.8º
ω scans	*h* = −7→7
Absorption correction: multi-scan(CrystalClear; Molecular Structure Corporation & Rigaku, 1999)	*k* = −9→9
*T*_min_ = 0.974, *T*_max_ = 0.991	*l* = −34→34
10940 measured reflections	

### Refinement

**Table:**

Refinement on *F*^2^	Secondary atom site location: difference Fourier map
Least-squares matrix: full	Hydrogen site location: inferred from neighbouring sites
*R*[*F*^2^ > 2σ(*F*^2^)] = 0.051	H-atom parameters constrained
*wR*(*F*^2^) = 0.135	*w* = 1/[σ^2^(*F*_o_^2^) + (0.0654*P*)^2^ + 0.4321*P*] where *P* = (*F*_o_^2^ + 2*F*_c_^2^)/3
*S* = 1.10	(Δ/σ)_max_ < 0.001
2724 reflections	Δρ_max_ = 0.32 e Å^−^^3^
155 parameters	Δρ_min_ = −0.39 e Å^−^^3^
Primary atom site location: structure-invariant direct methods	Extinction correction: none

### Special details

**Table:**

Geometry. All e.s.d.'s (except the e.s.d. in the dihedral angle between two l.s. planes) are estimated using the full covariance matrix. The cell e.s.d.'s are taken into account individually in the estimation of e.s.d.'s in distances, angles and torsion angles; correlations between e.s.d.'s in cell parameters are only used when they are defined by crystal symmetry. An approximate (isotropic) treatment of cell e.s.d.'s is used for estimating e.s.d.'s involving l.s. planes.
Refinement. Refinement of *F*^2^ against ALL reflections. The weighted *R*-factor *wR* and goodness of fit *S* are based on *F*^2^, conventional *R*-factors *R* are based on *F*, with *F* set to zero for negative *F*^2^. The threshold expression of *F*^2^ > σ(*F*^2^) is used only for calculating *R*-factors(gt) *etc*. and is not relevant to the choice of reflections for refinement. *R*-factors based on *F*^2^ are statistically about twice as large as those based on *F*, and *R*- factors based on ALL data will be even larger.

### Fractional atomic coordinates and isotropic or equivalent isotropic displacement parameters (Å^2^)

**Table:**

	*x*	*y*	*z*	*U*_iso_*/*U*_eq_	
O1	0.06468 (18)	0.38400 (14)	0.43568 (4)	0.0246 (3)	
C1	0.0999 (2)	0.37824 (18)	0.38417 (5)	0.0196 (3)	
C2	−0.0458 (2)	0.29441 (18)	0.34780 (5)	0.0209 (3)	
H2	−0.1824	0.2384	0.3578	0.025*	
C3	0.0118 (2)	0.29399 (18)	0.29665 (5)	0.0205 (3)	
H3	−0.0875	0.2366	0.2720	0.025*	
C4	0.2110 (2)	0.37525 (17)	0.28040 (5)	0.0188 (3)	
C5	0.3523 (2)	0.46050 (18)	0.31800 (5)	0.0207 (3)	
H5	0.4884	0.5177	0.3082	0.025*	
C6	0.2972 (2)	0.46277 (18)	0.36889 (5)	0.0217 (3)	
H6	0.3944	0.5223	0.3935	0.026*	
C7	0.2705 (2)	0.37168 (17)	0.22661 (5)	0.0191 (3)	
C8	0.4701 (2)	0.44705 (18)	0.20655 (5)	0.0203 (3)	
H8	0.5885	0.5082	0.2255	0.024*	
C9	0.4594 (2)	0.41280 (18)	0.15079 (5)	0.0193 (3)	
C10	0.6102 (3)	0.45594 (19)	0.11311 (5)	0.0234 (3)	
H10	0.7468	0.5203	0.1215	0.028*	
C11	0.5577 (3)	0.40313 (19)	0.06279 (6)	0.0251 (3)	
H11	0.6602	0.4309	0.0368	0.030*	
C12	0.3566 (3)	0.31006 (19)	0.05014 (5)	0.0246 (3)	
H12	0.3239	0.2747	0.0157	0.030*	
C13	0.2030 (3)	0.26833 (18)	0.08766 (5)	0.0220 (3)	
H13	0.0648	0.2064	0.0790	0.026*	
C14	0.2559 (2)	0.31909 (17)	0.13805 (5)	0.0188 (3)	
C15	0.1310 (2)	0.29032 (18)	0.18543 (5)	0.0197 (3)	
H15A	−0.0226	0.3461	0.1824	0.024*	
H15B	0.1118	0.1623	0.1919	0.024*	
C16	−0.1393 (3)	0.3029 (2)	0.45282 (6)	0.0311 (4)	
H16A	−0.1495	0.3227	0.4897	0.047*	
H16B	−0.1347	0.1755	0.4460	0.047*	
H16C	−0.2736	0.3548	0.4345	0.047*	

### Atomic displacement parameters (Å^2^)

**Table:**

	*U*^11^	*U*^22^	*U*^33^	*U*^12^	*U*^13^	*U*^23^
O1	0.0271 (6)	0.0282 (6)	0.0184 (5)	−0.0015 (4)	0.0008 (4)	0.0018 (4)
C1	0.0221 (7)	0.0180 (6)	0.0187 (6)	0.0040 (5)	−0.0003 (5)	0.0017 (5)
C2	0.0195 (7)	0.0196 (7)	0.0237 (7)	−0.0020 (5)	0.0006 (5)	0.0014 (5)
C3	0.0205 (7)	0.0188 (6)	0.0219 (7)	−0.0012 (5)	−0.0019 (5)	−0.0009 (5)
C4	0.0197 (7)	0.0141 (6)	0.0224 (7)	0.0037 (5)	0.0004 (5)	0.0003 (5)
C5	0.0179 (7)	0.0195 (7)	0.0247 (7)	−0.0006 (5)	0.0011 (5)	−0.0011 (5)
C6	0.0205 (7)	0.0204 (7)	0.0240 (7)	0.0004 (5)	−0.0025 (5)	−0.0020 (5)
C7	0.0216 (7)	0.0139 (6)	0.0220 (7)	0.0030 (5)	0.0012 (5)	−0.0006 (5)
C8	0.0181 (7)	0.0212 (7)	0.0215 (7)	−0.0014 (5)	−0.0001 (5)	−0.0021 (5)
C9	0.0193 (7)	0.0158 (6)	0.0225 (7)	0.0011 (5)	−0.0001 (5)	−0.0001 (5)
C10	0.0229 (7)	0.0209 (7)	0.0267 (7)	−0.0024 (6)	0.0026 (6)	0.0015 (5)
C11	0.0280 (8)	0.0241 (7)	0.0235 (7)	0.0019 (6)	0.0060 (6)	0.0053 (6)
C12	0.0311 (8)	0.0230 (7)	0.0198 (7)	0.0021 (6)	0.0006 (6)	0.0011 (5)
C13	0.0242 (8)	0.0200 (7)	0.0216 (7)	−0.0013 (6)	−0.0018 (5)	0.0005 (5)
C14	0.0191 (7)	0.0157 (6)	0.0216 (7)	0.0016 (5)	0.0002 (5)	0.0011 (5)
C15	0.0192 (7)	0.0185 (6)	0.0211 (6)	0.0008 (5)	−0.0008 (5)	−0.0004 (5)
C16	0.0332 (9)	0.0371 (9)	0.0234 (7)	−0.0048 (7)	0.0043 (6)	0.0049 (6)

### Geometric parameters (Å, °)

**Table:**

O1—C1	1.3724 (16)	C9—C10	1.391 (2)
O1—C16	1.4301 (19)	C9—C14	1.409 (2)
C1—C6	1.392 (2)	C10—C11	1.394 (2)
C1—C2	1.3963 (19)	C10—H10	0.9500
C2—C3	1.394 (2)	C11—C12	1.394 (2)
C2—H2	0.9500	C11—H11	0.9500
C3—C4	1.399 (2)	C12—C13	1.397 (2)
C3—H3	0.9500	C12—H12	0.9500
C4—C5	1.4078 (19)	C13—C14	1.3919 (19)
C4—C7	1.4645 (19)	C13—H13	0.9500
C5—C6	1.384 (2)	C14—C15	1.4829 (19)
C5—H5	0.9500	C15—H15A	0.9900
C6—H6	0.9500	C15—H15B	0.9900
C7—C8	1.419 (2)	C16—H16A	0.9800
C7—C15	1.4542 (19)	C16—H16B	0.9800
C8—C9	1.4785 (19)	C16—H16C	0.9800
C8—H8	0.9500		
			
C1—O1—C16	117.40 (12)	C9—C10—C11	118.88 (14)
O1—C1—C6	115.60 (12)	C9—C10—H10	120.6
O1—C1—C2	124.55 (13)	C11—C10—H10	120.6
C6—C1—C2	119.85 (13)	C12—C11—C10	120.83 (14)
C3—C2—C1	119.13 (13)	C12—C11—H11	119.6
C3—C2—H2	120.4	C10—C11—H11	119.6
C1—C2—H2	120.4	C11—C12—C13	120.59 (13)
C2—C3—C4	122.18 (13)	C11—C12—H12	119.7
C2—C3—H3	118.9	C13—C12—H12	119.7
C4—C3—H3	118.9	C14—C13—C12	118.78 (14)
C3—C4—C5	117.14 (13)	C14—C13—H13	120.6
C3—C4—C7	121.50 (12)	C12—C13—H13	120.6
C5—C4—C7	121.36 (13)	C13—C14—C9	120.54 (13)
C6—C5—C4	121.43 (13)	C13—C14—C15	130.85 (13)
C6—C5—H5	119.3	C9—C14—C15	108.60 (12)
C4—C5—H5	119.3	C7—C15—C14	106.02 (12)
C5—C6—C1	120.26 (13)	C7—C15—H15A	110.5
C5—C6—H6	119.9	C14—C15—H15A	110.5
C1—C6—H6	119.9	C7—C15—H15B	110.5
C8—C7—C15	109.64 (12)	C14—C15—H15B	110.5
C8—C7—C4	125.75 (12)	H15A—C15—H15B	108.7
C15—C7—C4	124.61 (13)	O1—C16—H16A	109.5
C7—C8—C9	107.34 (12)	O1—C16—H16B	109.5
C7—C8—H8	126.3	H16A—C16—H16B	109.5
C9—C8—H8	126.3	O1—C16—H16C	109.5
C10—C9—C14	120.36 (13)	H16A—C16—H16C	109.5
C10—C9—C8	131.24 (13)	H16B—C16—H16C	109.5
C14—C9—C8	108.40 (12)		
			
C16—O1—C1—C6	178.34 (12)	C7—C8—C9—C10	−179.34 (14)
C16—O1—C1—C2	−2.0 (2)	C7—C8—C9—C14	0.24 (15)
O1—C1—C2—C3	−178.41 (13)	C14—C9—C10—C11	−0.7 (2)
C6—C1—C2—C3	1.3 (2)	C8—C9—C10—C11	178.81 (14)
C1—C2—C3—C4	−0.1 (2)	C9—C10—C11—C12	0.6 (2)
C2—C3—C4—C5	−0.7 (2)	C10—C11—C12—C13	0.3 (2)
C2—C3—C4—C7	179.21 (13)	C11—C12—C13—C14	−0.9 (2)
C3—C4—C5—C6	0.4 (2)	C12—C13—C14—C9	0.8 (2)
C7—C4—C5—C6	−179.47 (13)	C12—C13—C14—C15	−178.47 (14)
C4—C5—C6—C1	0.7 (2)	C10—C9—C14—C13	0.0 (2)
O1—C1—C6—C5	178.17 (12)	C8—C9—C14—C13	−179.60 (12)
C2—C1—C6—C5	−1.5 (2)	C10—C9—C14—C15	179.45 (12)
C3—C4—C7—C8	−178.77 (13)	C8—C9—C14—C15	−0.18 (15)
C5—C4—C7—C8	1.1 (2)	C8—C7—C15—C14	0.09 (15)
C3—C4—C7—C15	1.7 (2)	C4—C7—C15—C14	179.70 (12)
C5—C4—C7—C15	−178.42 (13)	C13—C14—C15—C7	179.39 (14)
C15—C7—C8—C9	−0.20 (15)	C9—C14—C15—C7	0.06 (15)
C4—C7—C8—C9	−179.80 (12)		
